# Anti-feline immunodeficiency virus reverse transcriptase properties of some medicinal and edible mushrooms

**DOI:** 10.14202/vetworld.2020.1798-1806

**Published:** 2020-09-05

**Authors:** Supaphorn Seetaha, Siriluk Ratanabunyong, Lueacha Tabtimmai, Kiattawee Choowongkomon, Jatuporn Rattanasrisomporn, Khuanjarat Choengpanya

**Affiliations:** 1 Center for Advanced Studies for Agriculture and Food, Institute for Advanced Studies, Kasetsart University, Bangkok 10900, Thailand; 2 Department of Biochemistry, Faculty of Science, Kasetsart University, Bangkok 10900, Thailand; 3 Interdisciplinary Graduate Program in Bioscience, Faculty of Science, Kasetsart University, Bangkok 10900, Thailand; 4 Department of Companion Animal Clinical Sciences, Faculty of Veterinary Medicine, Kasetsart University, Bangkok 10900, Thailand; 5 Program in Applied Biology, Maejo University Phrae Campus, Phrae 54140, Thailand

**Keywords:** feline immunodeficiency virus, fluorescence spectroscopy, mushrooms, crude extracts, reverse transcriptase

## Abstract

**Background and Aim:**

Feline immunodeficiency virus (FIV) causes AIDS-like symptoms in domestic and wild cats. Treatment of infected cats has been performed using human anti-HIV drugs, which showed some limitations. This study aimed to determine the anti-FIV potential of some mushrooms.

**Materials and Methods:**

A total of 17 medicinal and edible mushrooms were screened to find their inhibitory effect against FIV reverse transcriptase (FIV-RT). Three solvents, water, ethanol, and hexane, were used to prepare crude mushroom extracts. Fluorescence spectroscopy was used to perform relative inhibition and 50% inhibitory concentrations (IC_50_) studies.

**Results:**

The ethanol extract from dried fruiting bodies of *Inonotus obliquus* showed the strongest inhibition with an IC_50_ value of 0.80±0.16 μg/mL. The hexane extract from dried mycelium of *I. obliquus* and ethanol and water extracts from fresh fruit bodies of *Phellinus igniarius* also exhibited strong activities with the IC_50_ values of 1.22±0.20, 4.33±0.39, and 6.24±1.42 μg/mL, respectively. The ethanol extract from fresh fruiting bodies of *Cordyceps sinensis*, hexane extracts from dried mycelium of *I. obliquus*, ethanol extracts of *Ganoderma lucidum*, hexane extracts of fresh fruiting bodies of *Morchella esculenta*, and fresh fruiting bodies of *C. sinensis* showed moderate anti-FIV-RT activities with IC_50_ values of 29.73±12.39, 49.97±11.86, 65.37±14.14, 77.59±8.31, and 81.41±17.10 μg/mL, respectively. These mushroom extracts show anti-FIV potential.

**Conclusion:**

The extracts from *I. obliquus*, *P. igniarius*, *C. sinensis*, and *M. esculenta* showed potential anti-FIV activity.

## Introduction

Feline immunodeficiency virus (FIV) is a lentivirus which causes AIDS-like symptoms in domestic and wild cats and hyenas. The genome size of FIV is approximately 9400 base pairs, including *pol*, *gag*, and *env* genes. These genes are responsible for expressing three major functions of FIV proteins, which are virion structural proteins, reverse transcriptase, and viral envelope glycoprotein, respectively [1]. The infected cats suffer from other opportunistic diseases such as stomatitis, dermatitis and gingivitis, as well as neuropathological disorders and malignancy [2]. FIV infects T-cells, monocytes, macrophages, and neural cells by binding to the CD134 receptor and CXCR4 coreceptor. After viral entry, its single-stranded RNA genome is converted into proviral double-stranded DNA by FIV reverse transcriptase (FIV-RT) enzyme before integrating into the host genome [1]. Therefore, FIV-RT is a core enzyme in viral replication and infection. Treatment of FIV-infected cats could be performed by inhibiting FIV-RT, for example, the use of nucleotide analog or non-nucleoside reverse transcriptase inhibitors [3,4].

FIV-RT is a heterodimeric protein consisting of two subunits, p51 and p66. The p66 subunit contains N-terminal RNA-dependent DNA polymerase (RDDP) and C-terminal RNaseH domains, while the p51 subunit contains only RDDP domain. The RDDP domain of both subunits contains four subdomains which are fingers, palm, thumb, and connection, but the arrangement of these subdomains is different [5]. The inhibition of RT has mainly focused on the catalytic site on the RDDP domain of p66 and the non-nucleotide binding pocket (NNBP) which is located approximately 10 Å from the catalytic site. Two types of drugs have been used to inhibit FIV-RT by binding to these pockets. The first type of inhibitors is nucleotide analog or nucleoside analog reverse transcriptase inhibitors (NtARTIs or NARTIs). These inhibitors are widely used as antiviral drugs to treat viral infections in both human and cats. They share structural similarities to the natural substrates of the enzyme, therefore, both inhibitors bind at the active site of FIV-RT. Zidovudine (3´-azido-2´,3´-dideoxythymidine or AZT) was the first antiviral drug used to treat HIV infection in humans, and it was then applied to cure FIV infection in cats [3]. In human, 1-5 mg/kg AZT was injected intravenously every 4-8 h for 2 weeks, and then, 2-10 mg/kg AZT was given orally for another 2 weeks. All doses used could elevate number of helper-inducer lymphocyte and patient’s weight [6]. In cats, AZT could inhibit FIV replication in Crandell-Rees feline kidney cells and could improve the clinical status of the infected cats [4,7]. Application of AZT on infected cats at a dose of 2.5 mg/kg of body weight every 12 h for 3 weeks could improve general clinical status and CD4/CD8 ratio at average degrees of 0.6 and 0.2, respectively [7]. Although AZT could inhibit FIV replication *in vitro* and could improve the CD4/CD8 ratio, long-term treatment of FIV infection in cats could cause FIV mutation [8]. Moreover, the viral load was slightly increased in some cats after discontinuing the treatment [8]. Other NARTIs introduced to treat FIV-infected cats were 9-(2-phosphonylmethoxyethyl) adenine (PMEA) which showed more potent anti-FIV-RT activity than AZT. However, PMEA showed more severe side effects than AZT, which limits the use of PMEA for the treatment of FIV infection in cats [7]. The second type of antiviral drug is non-nucleoside reverse transcriptase inhibitors (NNRTIs). NNRTIs are hydrophobic compounds with diverse structures [9]. The binding of NNRTIs at the NNBP, which is located in close proximity to the active site, causes conformational change of the catalytic site leading to inhibition of viral replication [10]. Nevirapine, is one of the FDA-approved NNRTIs anti-HIV drugs, has shown to be ineffective against FIV [11]. Thus, the finding of new NNRTIs against FIV-RT will be a major breakthrough for the treatment of FIV-infected cats.

Recently, researchers have become interested in using mushrooms as a source of biologically active compounds for medical uses such as anticancer, antiviral, immunomodulating, as well as hepatoprotective agents [12-15]. Hispolon, a bioactive compound isolated from *Inonotus obliquus*, was found to be a potential anti-oral cancer. It could suppress cell proliferation of human epidermoid KB cell with a 50% inhibitory concentrations (IC_50_) value of 4.62±0.16 μg/mL [12]. Ganoderic acid α, ganoderiol F, and ganodermanontriol from *Ganoderma lucidum* showed anti-HIV-1 activity by inhibiting HIV-1-induced cytopathic effects and HIV-1 protease [13]. β-Glucan from *Grifola frondosa* and *Lentinus lepideus* showed immunomodulating activities such as promoting mouse bone marrow cell growth and differentiating and increasing the number of granulocytes and myeloid progenitors [14]. The crude extracts from *G. frondosa* and *Lentinula edodes* can reduce the level of serum transaminases, which were produced from injured liver cells from paracetamol [15]. Thus, mushrooms are an excellent source of promising pharmaceutical agents.

In this study, the extracts of 17 medicinal and edible mushrooms were screened for anti-FIV-RT properties. The objective of this study was to determine the anti-FIV potential of some mushrooms.

## Materials and Methods

### Ethical approval

This work was approved by the Research Committee of Kasetsart University and Maejo University Phrae Campus and was performed under project ID of MJU. 2-61-009.

### Study period and location

The study was performed at Faculty of Veterinary Medicine and Faculty of Science, Kasetsart University from January to July 2019.

### Chemicals

All chemicals used were of analytical grade except ethanol and hexane, which were of reagent grade. The EnzChek® Reverse Transcriptase Assay Kit was purchased from Molecular Probes (USA). Efavirenz (EFV) was from the Government Pharmaceutical Organization (GPO) (Thailand).

### Cloning, expression, and purification of FIV-RT

The amino acid sequence of FIV-RT from PDB ID 5OVN was used to design its corresponding DNA sequence. As FIV-RT is a heterodimeric protein containing p66 and p51 subunits in which p51 is produced from proteolytic processing of p66, the amino acid sequence of p66 was used to generate the DNA sequence by submitting to the GenScript Express Gene Synthesis Service (GenScript, USA). Codon optimization was also performed to ensure high-protein expression in *Escherichia coli*. The *fiv-rt* gene was synthesized and cloned into pET15b at *Nde*I and *Xho*I restriction sites and the new recombinant plasmid was named pET15b_FIV-RTp66. For cloning of p51, the primers were designed with *Nde*I and *Xho*I restriction sites, and pET15b_FIV-RTp66 was used as a template. PCR reaction was performed with 5´ATTCATATGGCGCAGATTAGCG3´ forward primer and 5´ATCTCGAGTTATTTAATGTTCAGCG3´ reverse primer. The DNA fragment of p51 was also cloned into the pColdI vector at the same restriction sites. The recombinant plasmids containing *fiv-rtp51* genes were named pClodI_FIV-RTp51. Each plasmid was individually transformed into *E. coli* DH5α and BL21 (DE3) for maintaining DNA stock and protein expression, respectively. The FIV-RT subunits p51 and p66 were individually expressed in 1 L of LB broth containing 0.5 mM IPTG at 180 rpm, 16°C for 16-18 h. Then, the induced cells were harvested and pooled together before subjecting to protein purification using ultrasonication for cell lysis and using Ni-Sepharose and Resource S cation exchange chromatography for protein purification [16]. After protein purification, the heterodimer of FIV-RT (p66/p51) was obtained for further inhibitory assays.

### Mushroom samples and preparation of crude mushroom extracts

The fruiting bodies and mycelium of 17 edible and medicinal mushrooms, namely, *Agaricus blazei*, *Dictyophora indusiata*, *G. lucidum*, *Hericium erinaceus*, *Hypsizygus marmoreus*, *I. obliquus*, *Lentinus squarrosulus*, *L. edodes*, *Lentinus* TAFRS007, *Lentinus* TAFRS011, *Lentinus* TAFRS014, *Morchella esculenta*, *Cordyceps sinensis*, *Pleurotus sajor-caju*, *Pleurotus eryngii*, *Phellinus igniarius*, and *Tremella fuciformis* were collected from local markets in Thailand. The fruiting bodies or mycelia were cut into small pieces, ground, and lyophilized for 2 days. Then, the dried small pieces of mushrooms were ground into powder and divided into three parts. Each part was extracted with each solvent, distilled water (DW), and 99.5% ethanol and hexane. Ethanol and hexane extractions were carried out by incubating at 37°C in a shaker at 180 rpm overnight, while the DW extraction was performed at 25°C overnight in a shaker at the same rpm. After that, all crude extracts were filtered through Whatman no.1 filters and centrifuged at 7000 rpm, 4°C for 15 min to remove precipitate. The ethanol and hexane solvents were removed using a hot air oven at 80°C, and the DW was removed by lyophilization. The mushroom powder was redissolved in 100% dimethyl sulfoxide (DMSO) to a final concentration of 100 mg/mL. The crude extracts were kept at −20°C until used.

### Relative inhibition assay

EnzChek® reverse transcriptase assay kit (Molecular Probes, USA) was used in this study. The relative inhibition assay was performed using fluorescence spectroscopy as previously described [17]. Briefly, 2 μL of 10 mg/mL crude mushroom extract was added into each well of a 384-well plate containing 13 μL RT reaction buffer (50 mM TE pH 7.6, 2 mM DTT, 20% glycerol). Then, 5 μL of 50 nM purified recombinant wild-type FIV-RT was added into each well. The reaction was started by adding 5 μL of 1:400 primer/template substrate. The reaction was incubated at 25°C for 30 min and was stopped by adding 5 μL 0.2 M EDTA. After that, 40 μL of 1:700 Picogreen was added to the stopped reaction. The reaction was then incubated in the dark for 5 min and fluorescence was measured at an excitation wavelength of 485 nm and an emission wavelength of 535 nm. EFV was used instead of the mushroom extract for the positive control reaction. The reaction without inhibitor was used as a negative control. The relative inhibition was calculated using the formula: Relative inhibition (%) = [(FI_P_−FI_S_) × 100]/FI_P_, where FI_P_ and FI_S_ were fluorescence intensities of the positive control and mushroom reactions, respectively. Three independent experiments were performed. Mushrooms which showed relative inhibition higher than 70% were chosen for further IC_50_ assays.

### Determination of IC_50_ values

A two-fold serial dilution of the mushroom crude extract was performed and was then used as an inhibitor in IC_50_ assays. The 2 μL of each two-fold diluted mushroom extract was mixed with 13 μL RT reaction buffer in a 384-well plate. Then, 5 μL of 50 nM purified recombinant wild-type FIV-RT was added into each well. The reaction was started by adding 5 μL of 1:400 primer/template. The reaction was incubated, stopped and the fluorescence intensity was measured as previously described. The IC_50_ was calculated from the non-linear regression dose–response curve generated by the GraphPad Prism program (GraphPad Software Inc., San Diego, CA, USA). The IC_50_ value of EFV against FIV-RT was also determined.

## Results

### Cloning, expression, and purification of FIV-RT

**Figure-1:**
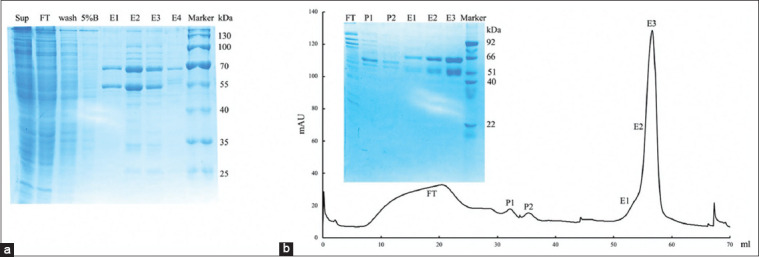
Purification of recombinant wild-type feline immunodeficiency virus reverse transcriptase. The enzyme was purified through Ni-Sepharose and determined with 12% SDS-PAGE (Sup=Supernatant, FT=Flow-through, Wash=Washed with buffer A, 5% B=Washed with 5% buffer B, E1-E4=Eluted fraction) (a). Then, resource S cation exchange chromatography was performed to obtained high purity protein and confirmed with 12% SDS-PAGE (FT=Flow-through, P1-P2=Peaks from 0% to 15% gradient buffer B, E1-E3=Elution peaks from 15% to 35% gradient buffer B) (b).

The amino acid sequence of FIV-RT from the PDB database under ID no. 5OVN was used for designing its corresponding DNA sequence. Codon optimization was also performed to ensure high-protein expression in the bacterial host. The DNA fragment of p66 was synthesized and cloned into the pET15b expression vector which was then used to generate a DNA fragment of p51. The p51 was also cloned into pColdI. Both subunits were individually transformed into *E. coli* BL21 (DE3) and expressed as intracellular His-tagged proteins. Purifications of both subunits were performed by combining IPTG-induced *E. coli* BL21 (DE3) cells harboring each construct before subjecting to sonication and Ni-Sepharose and Resource S cation chromatography. The final yield of purified recombinant wild-type FIV-RT was 8-10 mg from 1 L of culture medium. Each subunit of purified FIV-RT showed molecular weights of 66 and 51 kDa, respectively, on 12% SDS-PAGE as expected, and more than 90% purity of recombinant FIV-RT was obtained as judged by SDS-PAGE (Figure-1).

### Mushroom crude extracts

The 17 mushrooms used in this study were collected from local distributors in Thailand and were extracted with three different solvents. Sixty-four crude mushroom extracts were obtained. Each mushroom extract was dissolved in 100% DMSO to a final concentration of 100 mg/mL and kept at −20°C until used.

### Relative inhibition of FIV-RT

**Figure-2:**
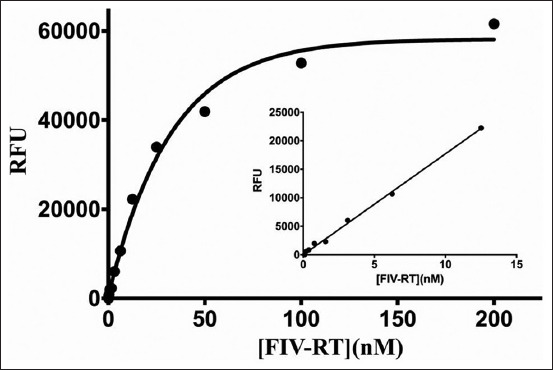
Feline immunodeficiency virus reverse transcriptase (FIV-RT) activity determination. The activity of different concentrations of purified recombinant wild-type FIV-RT was tested using the EnzChek® reverse transcriptase assay kit. A 10 nM of FIV-RT was used in the subsequent inhibition assays.

**Figure-3:**
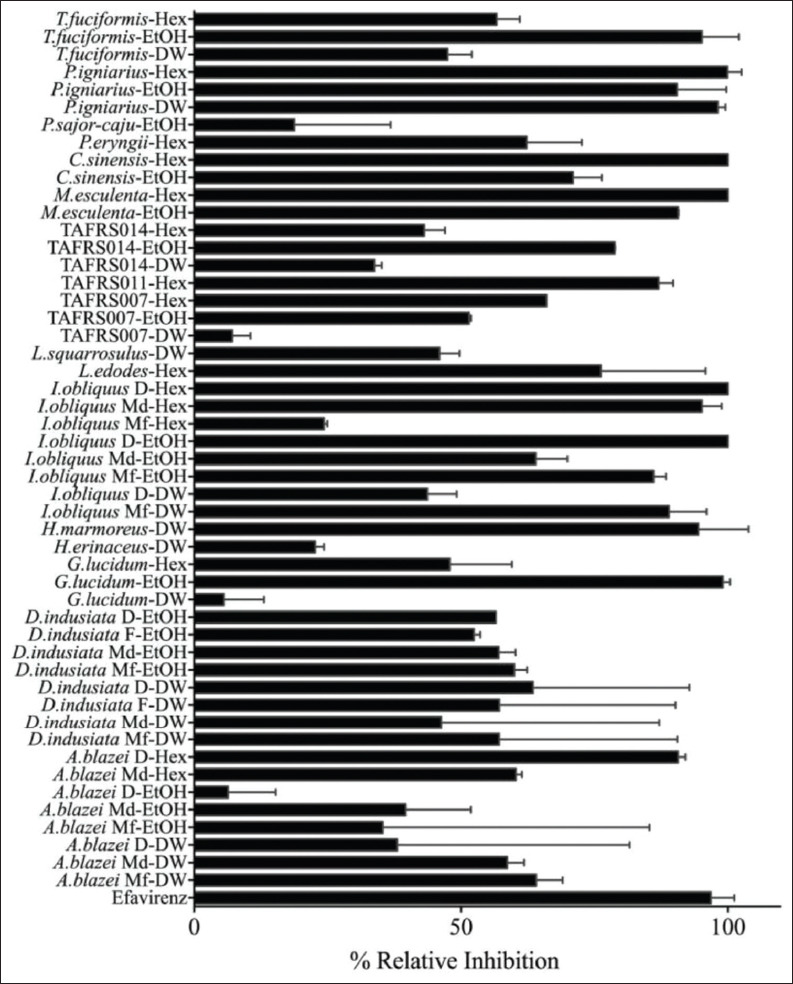
Relative inhibition of mushroom crude extracts. A final concentration of 0.8 mg/mL of each mushroom extracts was used.

To determine whether the purified recombinant wild-type FIV-RT was active or not, the activity of the enzyme was tested using the EnzCheck® Reverse Transcriptase Assay Kit. The different concentrations of the enzyme were reacted with substrate as described in the relative inhibition assay. The results showed that enzyme concentrations up to 15 nM were linearly proportional to relative fluorescence unit. Thus, the enzyme concentration at 10 nM was used in the subsequent experiments (Figure-2). The relative inhibition of FIV-RT by crude mushroom extracts was performed by incubating 0.8 mg/mL of each mushroom extract with 10 nM purified recombinant wild-type FIV-RT. Crude mushrooms extracts showed various inhibition activities against FIV-RT (Figure-3). The DW extracts of fresh fruiting bodies of *L. edodes*, *Lentinus* TAFRS011, *M. esculenta*, *C. sinensis*, *P. eryngii*, *P. sajor-caju*, and dried mycelium of *I. obliquus*; ethanol extracts of fresh fruiting bodies of *H. marmoreus*, *L. edodes*, *L. squarrosulus*, *P. eryngii*, and dried mycelium of *Lentinus* TAFRS011; and hexane extracts of fresh fruiting bodies of *H. marmoreus* and *P. sajor-caju* showed no inhibition activity against FIV-RT. The extracts which showed relative inhibition higher than 70% were selected for IC_50_ assay. Thus, the DW extracts of fresh fruiting bodies of *H. marmoreus* and *P. igniarius* and fresh mycelium of *I. obliquus*; ethanol extracts of fresh fruiting bodies of *G. lucidum*, *M. esculenta*, *C. sinensis*, *P. igniarius*, and *T. fuciformis*, dried fruiting bodies and fresh mycelium of *I. obliquus*, and dried mycelium of *Lentinus* TAFRS014; and hexane extracts of fresh fruiting bodies of *L. edodes*, *M. esculenta*, *C. sinensis*, and *P. igniarius*, dried fruiting bodies of *A. blazei* and *I. obliquus* and dried mycelia of *I. obliquus* and *Lentinus* TAFRS011 were selected for further study.

### IC_50_ value

**Figure-4:**
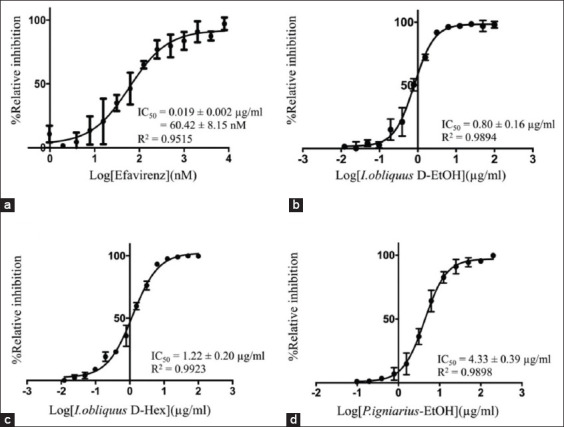
(a-d) The 50% inhibitory concentrations values of crude mushroom extracts which showed strong inhibitory activity against feline immunodeficiency virus reverse transcriptase.

The determination of the IC_50_ of crude mushroom extracts against FIV-RT was performed with the final concentrations of the extracts ranging from 0.012 μg/mL to 26 mg/mL, and the results are shown in Table-1 and Figure-4. The ethanol extract of dried fruiting bodies of *I. obliquus* showed the strongest anti-FIV-RT activity with an IC_50_ value of 0.80±0.16 μg/mL, while its hexane extract showed an IC_50_ value of 1.22±0.20 μg/mL. Other crude mushroom extracts, such as ethanol and DW extracts of *P. igniarius*, ethanol extract of fresh fruiting bodies of *C. sinensis*, hexane extract of dried mycelium of *I. obliquus*, ethanol extract of fresh fruiting bodies of *G. lucidum*, and hexane extracts of fresh fruiting bodies of *M. esculenta* and *C. sinensis* also exhibited strong inhibition against FIV-RT (Table-1). However, the IC_50_ values of these mushroom extracts were lower than that of EFV, which was 0.019±0.002 μg/mL. The hexane extract of fresh fruiting bodies of *P. igniarius* showed the weakest anti-FIV-RT activity with an IC_50_ value of 1.630±251.03 μg/mL.

**Table-1:** IC_50_ of crude mushroom extracts compared with efavirenz.

Sample	IC_50_ (mg/mL)
Efavirenz	0.019±0.002
*Agaricus blazei* D-Hex	502.60±126.82
*Ganoderma lucidum*-EtOH	65.37±14.14
*Hypsizygus marmoreus*-Hex	715.60±257.97
*Inonotus obliquus* Mf-DW	852.80±350.56
*Inonotus obliquus* Mf-EtOH	391.70±127.50
*Inonotus obliquus* D-EtOH	0.80±0.16
*Inonotus obliquus* Md-Hex	49.97±11.86
*Inonotus obliquus* D-Hex	1.22±0.20
*Lentinula edodes*-Hex	477.80±132.08
TAFRS011-Hex	1.727±442.04
TAFRS014-EtOH	317.20±66.72
*Morchella esculenta*-EtOH	211.90±64.25
*Morchella esculenta*-Hex	77.59±8.31
*Cordyceps sinensis*-EtOH	29.73±12.39
*Cordyceps sinensis*-Hex	81.41±17.10
*Phellinus igniarius*-DW	6.24±1.42
*Phellinus igniarius*-EtOH	4.33±0.39
*Phellinus igniarius*-Hex	1630.00±251.03
*Tremella fuciformis*-EtOH	623.70±253.64

IC_50_=50% Inhibitory concentrations

## Discussion

FIV causes AIDS-like symptoms in cats. The infected cats suffer from various opportunistic infectious diseases. Moreover, FIV infection is persistent and lifelong treatment is required which will be costly for the cat owners. The use of human anti-HIV drugs showed a promising result; however, it showed some limitations such as drug side effects, reappearance of virus after discontinuing treatment, and occurrence of mutant FIV [7,8]. Thus, it is necessary to search for new medicine to treat the infected cats. Mushrooms have gained more attention in the past few years due to their abundance of bioactive compounds [12-15]. In this study, 17 mushrooms were chosen to screen for anti-FIV-RT activity.

Cloning, expression, and purification through Ni-Sepharose and resource S cation exchange chromatography yielded an active dimeric FIV-RT, which was then used for screening of anti-FIV-RT agents. The inhibition studies revealed that the extracts from *I. obliquus* showed strong inhibitory activity against FIV-RT with IC_50_ values of 0.80±0.16 and 1.22±0.20 μg/mL for *I. obliquus* D-EtOH and *I. obliquus* D-Hex, respectively. *I. obliquus* is classified as a medicinal mushroom and has been used in traditional medicine for the prophylaxis and treatment of various diseases such as gastric disorder, diabetes mellitus, cancers, cardiovascular, and viral infection diseases [12,18-25]. The water/ethanol extract of *I. obliquus* showed anti-herpes simplex virus (HSV) type 1 activity in infected Vero cells [21,22]. The extract showed the IC_50_ of 3.82 and 12.29 μg/mL in the plaque reduction and HSV-1/blue assays, respectively [21]. The 5 μg/mL rate of *I. obliquus* extract could protect the infected cells from HSV-induced cytodestruction. The best protective effect was observed when *I. obliquus* extracts were added before or within 1 h after HSV infection [22]. The mechanism of anti-HSV was found to be at the initial stage of viral infection by inhibiting membrane fusion between HSV and Vero cells. In addition, *I. obliquus* extract showed anti-hepatitis C virus, human influenza viruses A and B, and human immunodeficiency virus type 1 activity in cell-based assays [20,23]. Chemical analysis of the methanol extract of *I. obliquus* revealed that it contains protocatechuic acid which has been shown to have antioxidant, antibacterial, antiviral, and immune regulating activities [19]. Moreover, Tian *et al*. [24] stated that polysaccharides from *I. obliquus* (IOPs) could act as a broad-spectrum antiviral drug against feline calicivirus, feline herpesvirus 1, feline influenza virus H3N2 and H5N6, feline panleukopenia virus, and feline infectious peritonitis virus by blocking viral binding/absorption and replication. From these studies, the bioactive compounds responsible for anti-FIV-RT activity in *I. obliquus* extract might be protocatechuic acid or IOPs. The solvents and methods used to obtain the extracts from mushrooms could affect the chemical constituents in the extracts [19]. Therefore, it is mandatory that further studies will be performed to clarify whether these compounds or other specific components are responsible for anti-FIV-RT property of *I. obliquus*.

The extracts from *P. igniarius* also exhibited strong anti-FIV-RT activity with the IC_50_ values of 4.33±0.39 μg/mL and 6.24±1.42 μg/mL for *P. igniarius*-EtOH and *P. igniarius*-DW, respectively. *P. igniarius* is also classified as a medicinal mushroom and is one of the most used mushrooms in Asian folk medicine to treat many diseases, including cancers. Lee *et al*. [26] reported that the water extract of *P. igniarius* had antiviral activity against influenza A and B, including pandemic H1N1, human H3N2, avian H9N2, and oseltamivir-resistant H1N1 viruses by blocking virus attachment to the cell surface receptor. Lee *et al*. also speculated that its polysaccharides might be responsible for the antiviral activity as they are the main components of the mushroom extracts [26]. The water extract of *P. igniarius* also showed anti-HSV-1 activity in Vero cells [27]. The study of the chemical composition revealed that the mushroom contains hispidin, which showed anti-influenza viruses H1N1 and H3N2 and anti-HIV-1 integrase activity. Hispidin is of interest to use in drug discovery [28]. Again, it is necessary that further studies be performed to evaluate the effect of hispidin on FIV-RT activity and search for novel active compounds against FIV-RT.

The extracts of *G. lucidum*, *M. esculenta*, and *C. sinensis* also showed good anti-FIV-RT activity. They could inhibit FIV-RT at a similar strength (IC_50_ values ranging from 29.73±12.37 to 81.41±17.10 μg/mL, which were approximately 10-fold higher IC_50_ values than those of *I. obliquus* and *P. igniarius*). The extract of *G. lucidum* showed a broad spectrum of antiviral activity against various viruses such as *Enterovirus* 71, hepatitis B virus, HIV-1, HSV-1, and vesicular stomatitis virus. These antiviral activities were mainly from polysaccharides and triterpenes [29]. *C. sinensis* and *M. esculenta* also exhibited wide range of pharmacological properties including antiviral, antimicrobial, antioxidant, antitumor, anti-inflammatory, and immune regulation [30]. Research on the antiviral activity of *C. sinensis*, has been conducted. In addition, Ohta *et al*. [31] found that acidic polysaccharide (APS) extracted from *Cordyceps militaris* could reduce influenza A titer in infected mice. APS could also modulate the immune function of macrophages and increase mice survival rate. Cordycepin, adenosine analog (3′-deoxyadenosine), and its derivatives showed anti-influenza, plant, HIV, murine leukemia, and Epstein–Barr viruses activity [32,33]. In the case of *M. esculenta*, the study on antiviral activity of this mushroom is limited. To the best of our knowledge, antiviral activity of *M. esculenta* has not been previously reported. This study was the first on the antiviral property of *M. esculenta* extract. Further research is also required to separate and identify bioactive compounds responsible for anti-FIV-RT properties.

Comparison of the IC_50_ values of these mushroom extracts and anti-HIV drug EFV revealed that the IC_50_ values of mushroom extracts were much higher than that of EFV. However, this study clearly showed the anti-FIV potential of these mushroom extracts through inhibition of the RT enzyme which is important for viral infection. Further study is needed to investigate whether the anti-FIV activity is due to a specific component or the combined effect of various individual constituents. As mushroom extracts have been shown to possess antioxidant activity, they can be used as a tonic to reduce oxidative stress during acute FIV infection [34]. Moreover, mushrooms have been reported to show immunomodulating activity. They could be used in combination with drugs to initiate and support immune responses [31,35]. Synergism between a mushroom extract and antiviral drug has also been reported [36]. It is noteworthy that extraction with nonpolar solvents (hexane and ethanol) gave extracts with great inhibitory activity against FIV-RT. The chemical constituents in these extracts might be hydrophobic compounds. NNRTIs are small hydrophobic molecules with diverse structures. Thus, these mushroom extracts may contain novel NNRTIs. The water extract also showed good anti-FIV-RT activity. It has been reported that some proteins and polysaccharides extracted from mushrooms showed antiviral potential [12,24,25,27,29,31,32,35,37,38] (Table-2). Thus, the experimental design to obtain the anti-FIV compounds (e.g., type of inhibitor, solvent used, and purification method) should be taken into account in further studies and clinical applications.

**Table-2:** Possible bioactive substances, potential activity, and toxic effect in mushroom used in this study.

Mushroom	Bioactive substance	Potential activity	Toxic effect	References
*Cordyceps sinensis*	Cordycepin	Anti-herpes simplex virus type-1 activity in Vero cells	0.0005 mM	[32]
*Inonotus obliquus*	Hispolon	Inhibition of human epidermoid KB cell proliferation	IC_50_=4.62 mg/mL	[12]
	Inonotusol G Protocatechuic aldehyde 4-(3,4-Dihydroxyphenyl) but-3en-2-one 5-fluorouracil	Cytotoxicity against BEL7402, A-549, and KB human cell lines	IC_50_=3.1-9.9 mg/mL	[25]
	Protocatechuic acid	Antivirus		[37]
	Polysaccharide	Anti-virus against feline herpes virus, feline influenza virus, feline panleukopenia virus, and feline infectious peritonitis virus	IC_50_=18.15-68.47 mg/mL	[24]
	Polysaccharide	Anti-HIV-1 protease	IC_50_=2.5 mg/mL	[29]
*Phellinus igniarius*	Hispidin	Anti-HIV-1 integrase	2 mM	[27]
	Phelligridin D	Antiviral activity against influenza A virus H1N1 in MDCK cells	IC_50_=24.6 mM	[38]
*Ganoderma lucidum*	Ganoderiol F	Inhibition of HIV-1-induced cytopathic effect in MT-4 cells	7.8 mg/mL	[29]
	Ganoderic acid B	Anti-HIV-1 protease	0.17 mM	[29]

## Conclusion

A total of 17 medicinal and edible mushrooms widely distributed in Thailand were chosen to screen for anti-FIV-RT activity. Comparison of the inhibition potentials of these mushrooms against FIV-RT revealed that the ethanol extract from dried fruiting bodies of *I. obliquus* showed the strongest inhibition activity with an IC_50_ value of 0.80±0.16 μg/mL. The hexane extract from dried mycelium of *I. obliquus* and ethanol and water extracts from fresh fruiting bodies of *P. igniarius* also exhibited strong activities with the IC_50_ values of 1.22±0.20, 4.33±0.39, and 6.24±1.42 μg/mL, respectively. The ethanol extract from fresh fruiting bodies of *C. sinensis*, hexane extract from dried mycelium of *I. obliquus* and fresh fruiting bodies of *G. lucidum*, and the hexane extract of fresh fruiting bodies of *M. esculenta* and *C. sinensis* showed moderate anti-FIV-RT activities with IC_50_ values of 29.73±12.39, 49.97±11.86, 65.37±14.14, 77.59±8.31, and 81.41±17.10 μg/mL, respectively. These mushroom extracts show anti-FIV potential. However, further study is required to identify the specific constituents responsible for anti-FIV-RT activity and their mechanism of action. In addition, the studies on the immune effect and interaction between mushroom extracts and anti-FIV drug candidates are also required for its clinical application.

## Authors’ Contributions

JR: Principle investigator, designed the study, drafted and critically revised the manuscript. KhCh: A research coordinator, performed cloning of FIV-RT and inhibition experiments, drafted and critically revised the manuscript. SS performed expression and purification of FIV-RT and sample collection, drafted and revised the manuscript. KC participated in design of the study and interpretation of the data. SR and LT performed mushroom extraction. All authors read and approved the final manuscript.

## Data Availability

Supplementary data can be available from the corresponding author.
